# An Effectiveness Assessment of China’s WEEE Treatment Fund

**DOI:** 10.3390/ijerph15051028

**Published:** 2018-05-19

**Authors:** Wenyan Zhao, Jianxin Yang

**Affiliations:** 1 State Key Laboratory of Urban and Regional Ecology, Research Center for Eco-Environmental Sciences, Chinese Academy of Sciences, Beijing 100085, China; zhaowy0928@sina.com; 2 College of Resources and Environment, University of Chinese Academy of Sciences, Beijing 100049, China

**Keywords:** WEEE, WEEE treatment fund, effectiveness assessment, evaluation indicator

## Abstract

Policy is essential to the management of waste electrical and electronic equipment (WEEE). In order to present valuable findings for policy improvement, we performed a quantitative effectiveness assessment of China’s WEEE treatment fund from 2012 to 2015. The achievement of the general goal of the WEEE treatment fund was evaluated with various indicators. We calculated the values of all indicators and analyzed the changes in them. The results show that the WEEE treatment fund was important in promoting WEEE collection and recycling activities and has provided great benefits in the form of resources and the environment. Moreover, the authorized enterprises also experienced progress in their development. In a word, the WEEE treatment fund was effective to some extent. However, because of the limited subsidies and other factors, the WEEE treatment fund had different effects on five categories of WEEE. We found that its approach worked best for the TV set. Although the promotional effects on the other four categories of WEEE have been increasingly significant since 2014, there is room for improvement. Fortunately, the subsidy rates have been adjusted by administrations and new subsidies were provided in 2016. As it is crucial for the effectiveness of the WEEE treatment fund, new subsidy rates should be evaluated regularly.

## 1. Introduction

WEEE has been an object of worldwide concern due to the variety of hazardous substances and recyclable resources it contains [1,2,3]. Many developed countries, especially European countries, have released different incentive policies such as fund subsidies based on the extended producer responsibility (EPR) [4] to promote the recycling of WEEE [5,6]. The WEEE recycling industry in China also expanded gradually due to the huge demand for recovered materials. There are two types of recyclers in this industry, namely, authorized and informal. The authorized enterprises perform better in the aspects of resources and the environment because of the advanced equipment and normative operation. However, the informal recyclers dispose of WEEE in a primitive and hazardous manner without treatment for pollution, meaning the recycling activities in informal sectors always cause severe contamination [7].

The WEEE recycling industry in China has always been dominated by informal recyclers [8,9,10], which means that most WEEE are not recycled in the environmentally friendly and resource-efficient manner [11,12,13,14]. This is mainly due to the fact that the WEEE recyclers have to pay for the WEEE collected, and the informal recyclers have a cost advantage for their low level of investment. Therefore, although a number of authorized enterprises were established in the past decade, they are facing severe challenges because there is not enough WEEE to meet their demand.

In order to promote efficient collection and recycling activities in regulated channels, China’s WEEE treatment fund was released in 2012. The fund adopts the principle of EPR to regulate the WEEE flow direction actively. It requires the producers and importers to fulfill their financial responsibility for the collecting and recycling of WEEE in the form of paying subsidies. These subsidies will then be provided to authorized enterprises to help them enhance their competitiveness, so that more WEEE will get standardized treatment in the authorized enterprises, rather than causing pollution in informal sectors. At present, a total of 109 recycling enterprises have been authorized by the administration. However, the WEEE treatment fund is currently only valid for the TV set (TV), refrigerator (RE), washing machine (WM), air-conditioner (AC), and personal computer (PC). These electronic items form the five categories of WEEE. The surcharge rates of electrical and electronic equipment (EEE) and the subsidy rates of WEEE can be found in Table 1. It should be noted that the subsidy rates have been adjusted and graded according to different specifications of WEEE since 2016.

The WEEE treatment fund has made progress in the past five years, and it experienced problems that resulted in adverse impacts on its mission as well. However, there was little systematical research on the effectiveness assessment of the WEEE treatment fund, especially for the quantitative research. In consideration of the fact that the WEEE treatment fund aims to promote both the collection and recycling activities and achieve great benefits of resource and environment, we developed a framework based on a set of indicators in the aspects of processing and performance respectively. On the basis of the calculation of the values of all indicators and the analysis of the changes in them, we assessed the effectiveness of the WEEE treatment fund from 2012 to 2015, so as to provide useful information and suggestions for the revision of this policy. It can be concluded that the WEEE treatment fund has played a significant role in promoting collection and recycling activities and gained great benefits in the form of resources and the environment, but the promotional effects on five categories of WEEE were different due to the limited subsidies.

## 2. Methods

### 2.1. Indicator Development

The effectiveness assessment evaluated the achievement of the objectives of the WEEE treatment fund. The general objective of the WEEE treatment fund is to promote normative collection and recycling activities through providing the subsidies to authorized recycling enterprises. As so many valuable resources can be obtained from the normative recycling processes and these activities in authorized enterprises are more environment-friendly compared to those in informal sectors in general, the WEEE treatment fund can further improve the resource efficiency and eliminate environmental pollution in the recycling processes. Based on these goals, the effectiveness assessment was derived from two aspects. First, it assessed the efficiency of the waste stream, and second, it evaluated the benefits of the resources and the environment.

In order to investigate the WEEE stream change in the implementation of the WEEE treatment fund, the assessment was divided into two phases: the collection and recycling with consideration to the WEEE stakeholders. The collected quantity (CQ) and collection rate (CR) were selected as the indicators for collection. CQ refers to the quantity of WEEE collected by the recyclers, which can determine the efficiency of the WEEE treatment fund in diverting more WEEE to authorized recyclers. CR refers to the share of CQ in the total quantity of WEEE, which was used to assess the function of the WEEE treatment fund in promoting the collection of WEEE. The total quantity of WEEE is the sum of the theoretical estimation of WEEE in the given year and the WEEE stocks in society.

The indicators for the recycling processes included the normative recycled quantity (NRQ), normative recycling rate (NRR), and recycling capacity rate (RCR). The NRQ was used as the valuation of the function of the WEEE treatment fund to promote normative recycling activities. NRR is the share of NRQ in the total quantity of WEEE, which was used to review the performance of the WEEE treatment fund in the promotion of recycling activities and the potential for improvement. RCR is the ratio of the NRQ to the recycling capacity, which was used in the evaluation of the WEEE treatment fund’s ability to help the authorized recyclers improve their recycling performance.

To evaluate the resource and environmental benefits of the WEEE treatment fund, the resources recovered through the collection and recycling processes were calculated. The resource performance was evaluated using the recovered resources (RR) and recovered resource rate (RRR). RR refers to the total sum of the materials recovered from WEEE and should be calculated based on the average level of the materials recovered from the given WEEE. RRR is the share of RR in the total weight of WEEE, which was used to assess the function of the WEEE treatment fund in the promotion of resource recovery.

As mentioned above, the environmental performance of authorized recyclers is much better than that of informal recyclers. Thus, the WEEE treatment fund promotes the disposal activities in formal channels, which is equivalent to reducing the possibility of WEEE recycling in informal channels and reducing the environmental risk in recycling processes. Based on this, the indicator “environmental performance” refers to the reduction potential of the environmental impact of the collection and recycling of WEEE. For this purpose, the Life Cycle Assessment (LCA) tool [15,16,17] was employed to quantify and compare the environmental impact from the authorized recyclers and informal recyclers.

### 2.2. Indicator Explanations and Data Sources

#### 2.2.1. Computational Methods

The indicators and corresponding computational methods are described in Table 2.

In Table 2, the data for C_n_ was retrieved from the recycled WEEE database built by the Ministry of Environmental Protection (MEP), and were originally reported by the 109 authorized recyclers. We assume that all WEEE entering the authorized enterprises will be recycled instantly, with no stock remaining. The data for D_n_ and P_n_ were also taken from the MEP database mentioned above. More specifically, in order to obtain the data for D_n_, the MEP will further check and review the data reported by the authorized recyclers (namely the data for C_n_) and eliminate unreasonable parts. Therefore, the values of D_n_ were generally less than those of C_n_. However, under the guidance of the WEEE treatment fund, the authorized recyclers were more familiar with the standardized operation, which led to the situation in which the gap between C_n_ and D_n_ narrowed gradually. The data for f_i_ and $\bar{w}$, which were used to estimate the recovered resources, were collected based on the practices of several authorized enterprises, including TES-AMM (Guangzhou) Co., Ltd. and Li Tong Group (LTG) in Guangdong Province (see supplementary files Tables S1 and S2) . The resources recovered from five categories of WEEE generally include iron and its alloys, copper and its alloys, aluminum and its alloys, nickel, gold, glass, plastic. Only the total quantity of WEEE (Q_n_′) and the environmental impact need to be calculated separately.

#### 2.2.2. Estimation of Total Quantity of WEEE

The total quantity of WEEE is the sum of the stock in society and the theoretical estimation of WEEE. As the WEEE treatment fund was implemented in 2012, the collection and recycling activities under the framework of the fund were absent before 2012. As a result, we set 2012 as the initial year and the total quantity of WEEE in 2012 is the sum of the stock in society in 2012 and the theoretical estimation of WEEE in 2012. However, the stock in society in 2013–2015 is equal to the total quantity of WEEE in the previous year minus the collected quantity in the previous year. The equation is as follows:

(1) $$\{\begin{array}{c} Q_{n}^{\prime }={\mathrm{St}}_{0}+Q_{n}\;,\;\left(\mathrm{year}\;2012\right)\\ Q_{n}^{\prime }=Q_{n-1}^{\prime }-C_{n-1}+Q_{n},\left(\mathrm{year}\;2013-2015\right) \end{array}$$

where Q_n_′ is the total quantity of WEEE; St_0_ is the WEEE stock in society in 2012; Q_n_ is the theoretical estimation of WEEE; and C_n−1_ is the collected quantity. As a matter of fact, it is very difficult to obtain the data for WEEE stocks in society. However, because the key factor to the effectiveness assessment of the WEEE treatment fund is the change of the collection rate, St_0_ can be assumed to be 0. And the data for the theoretical WEEE estimation (Q_n_) can be calculated using Equation (2).

The theoretical estimation of WEEE refers to the quantities of WEEE that are no longer suitable or able to be used for various reasons. The estimation methods can be divided into three categories, including (1) methods based on the product’s characteristics (here, mainly the lifetime); (2) methods based on the law of the conservation of matter; and (3) methods based on mathematical modeling [18,19,20,21,22,23,24,25]. According to the analysis of the characteristics of various estimation methods, such as data requirements, advantages and disadvantages, limitations and source of errors, the “market supply A” method [19] was selected (Equation (2)).

(2) $$Q_{n}=\sum \left(S_{i}\times p_{i}\right)$$

where S_i_ is the sale of EEE in year *n-i*; p_i_ is the lifespan distribution of EEE; *i* is the lifespan of EEE; and *n* is a given year.

Using the assumptions about the lifespan of EEE proposed by several experts [26,27,28,29,30,31], the lifespans of the TV set, refrigerator, washing machine, air-conditioner, and personal computer were identified as 8–10 years, 8–12 years, 8–12 years, 7–11 years, and 2–6 years, respectively. Then we determined the lifespan distribution of five categories of WEEE, which was supposed to obey the normal distribution (see supplementary files Table S3).

Since the statistical data for the sales of EEE were not available, they should be estimated using Equation (3).

(3) $$S_{i}=P_{i}+I_{i}-E_{i}$$

where P_i_, I_i_, and E_i_ are the production, import, and export of EEE in year *n-i*, respectively; *i* is the lifespan of EEE. These data were taken from the China Statistical Yearbook and China Customs Statistical Yearbook. Because of the differences in their lifespans, the data for the sales of five categories of EEE were from different years. The sales of EEE and the theoretical estimation of WEEE are presented in supplementary files Tables S4 and S5 respectively.

#### 2.2.3. Estimation of the Environmental Impact

• Goal and scope

**Figure 1:**
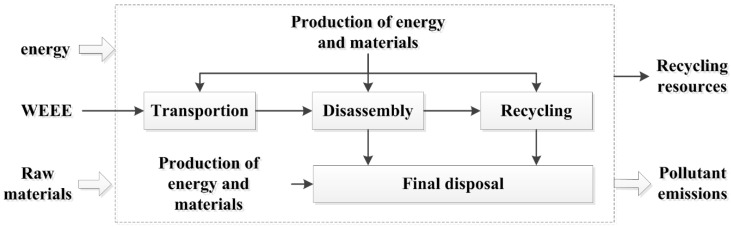
The system boundary.

With the support of LCA, the goal of this research was to learn about the environmental performance of the collection and recycling activities carried out by the authorized and informal recyclers. The system boundary is represented in Figure 1. Due to the diversification of the collection approaches, along with the complexity of the collectors [1], only the transportation between the WEEE owners and the WEEE recyclers was included in the system boundary (with respect to the collection stage). Since we were limited by the data available, the following processes were also excluded from the system boundary: the transportation of WEEE in recycling processes, the transportation of solid wastes after recycling, and the related infrastructure construction and maintenance. The functional unit was defined as one unit of one kind of WEEE that contained TV set, refrigerator, washing machine, air-conditioner, or personal computer.

• Life cycle inventory (LCI)

Both the input and output data are required for the LCI. For this research, the input data contained both the direct and indirect energy consumption and material consumption in the recycling processes. The output data contained the products of recycling and the direct pollution emitted to the atmosphere, water, and soil from the recycling processes, as well as the indirect emissions from the production of the energy and materials consumed in the recycling processes.

The process data of several representative authorized enterprises were synthesized to meet the data requirements of LCI. The data sources are described in detail in Table 3 and the recycling process data can be found in supplementary files Tables S6–S9. We compared our results with the results of the technology investigation carried out by the China Household Electric Appliance Research Institute (CHEARI) [32] and confirmed that the depth of processing, technology, and treatment method of the controlled components in the above-mentioned authorized enterprises that provided the basic data were consistent with those of most domestic authorized enterprises. In summary, the data adopted in this research were representative of the WEEE recycling industry. Besides, since the data were derived from the environmental impact reports or technical evaluation reports of typical authorized recycling, they were deemed to be credible in regards to reflecting the level of technology of the authorized enterprises.

In contrast, it is difficult to collect the data about the informal recyclers due to the lack of governance. To this end, the following assumptions [11] were made in consideration of the recycling processes of the authorized enterprises and the actual situations in informal sectors. First, it was assumed that the recycling processes of the informal recyclers were the same as those of the authorized enterprises, but some relevant equipment was removed. Second, it was assumed that the recovery rates of the informal recyclers were equal to those of the authorized enterprises. Third, it was assumed that all kinds of pollutants generated in the collection and recycling activities in informal channels were emitted into the environment directly without any treatment. Based on these hypotheses, the modified data of the authorized enterprises were used to simulate the conditions of the informal recyclers.

Furthermore, due to the emergence of urban agglomerations, the surrounding informal recyclers also developed rapidly to meet the growing demand for WEEE collection and recycling activities [10,37,38,39,40]. For this reason, some WEEE was recycled by the informal recyclers near the urban agglomeration. Therefore, the distances between the gathering places of the informal recyclers and the major cities nearby in the corresponding urban agglomeration, such as the Yangtze River Delta urban agglomeration, Pearl River Delta urban agglomeration, and Beijing-Tianjin-Hebei agglomeration, were collected with the help of Google Maps. We found that most of the informal recyclers were at a distance of 400–600 km from the cities, while the average distance between consumers and authorized enterprises was 500 km (based on a field investigation). To highlight the differences in the environmental performance of the recycling activities in the back-end stage between the authorized and informal recyclers, the average distance between the consumers and the informal recyclers was also identified as 500 km.

After the data collection, we built the LCI models of five categories of WEEE in Simapro (V8.2). We obtained the life cycle inventories, which consisted of the energy consumption (typically referring to the energy consumption of transportation and power consumption of equipment), material consumption, and the pollutant emissions to the atmosphere, water, and soil. Due to the large quantity of data, it was not possible (or necessary) to list the data individually.

The results of Life cycle impact assessment (LCIA) were calculated at the endpoint level using the ReCiPe (v1.04) method. In order to eliminate the barriers of method selection in LCIA, ReCiPe, which integrates the existing midpoint approach and endpoint approach, was created on the basis of the Eco-indicator99 method and CML2001 method [41,42,43]. At the midpoint level, 18 impact categories related to different environmental issues are addressed with associated sets of characterization factors. At the endpoint level, the impact categories mentioned above are converted and aggregated into three endpoint categories further, which consists of damage to human health, ecosystem diversity and resource availability. Weighting factors are also provided for these damage categories, and a single score can be obtained by aggregating the weighting results. We chose this method because it takes into consideration a broad set of impact categories so that the environmental impact can be fully realized. Moreover, the Eco-indicator99 method and CML2001 method have both been widely applied in LCA analysis [44].

## 3. Results and Discussion

### 3.1. Processing Aspect

#### 3.1.1. Collection Target

• Collected quantity

**Figure 2:**
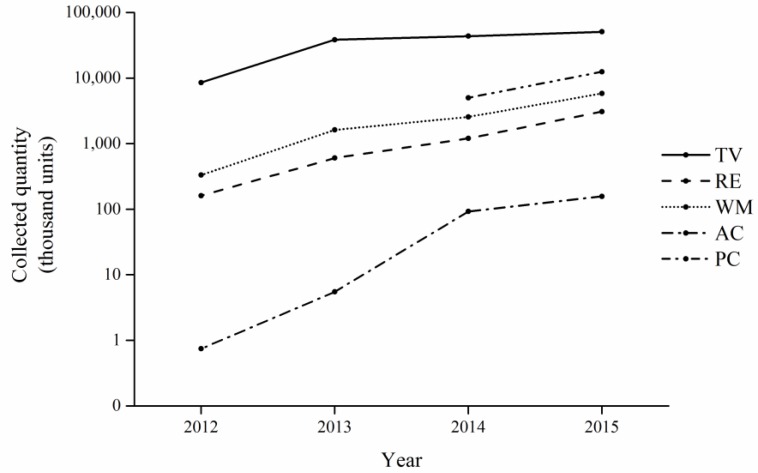
Waste electrical and electronic equipment (WEEE) delivered to the authorized recyclers from 2012 to 2015.

The quantities of WEEE collected by the authorized recyclers from 2012 to 2015 are shown in Figure 2.

**Figure 3:**
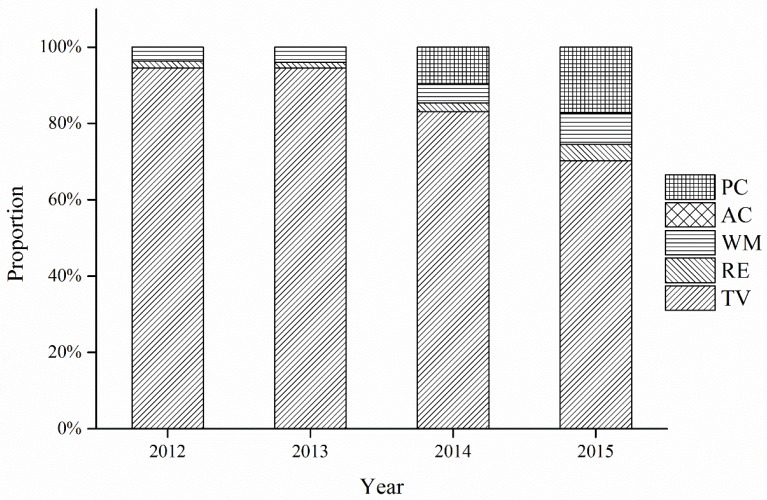
The proportion of five categories of WEEE in the total collected quantities from 2012 to 2015.

Figure 2 shows that the collected quantities of five categories of WEEE from 2012 to 2015 all showed a general uptrend. Moreover, the proportion of five categories of WEEE is shown in Figure 3. We can see from Figure 3 that the TV set dominated the five categories from 2012 to 2015, which means that the WEEE treatment fund has played the most significant role in promoting the collection and recycling of TV sets. This trend was due to the high subsidy rate for TV sets (85 yuan/unit [45]). In contrast, the other four categories of WEEE collected only accounted for a small share of the total quantities of WEEE collected. The air-conditioner comprised the smallest percentage, less than 1% during the time period studied. This situation may be due to the fact that the subsidy rate for air-conditioners (35 yuan/unit) is much lower than its collection price (263 yuan/unit [45]). Consequently, the authorized recyclers had little interest in collecting air-conditioners, so they were mainly delivered to the informal sectors, like the second-hand market, or informal recyclers. Accordingly, the subsidy rate for WEEE can influence the collection system significantly.

Besides, the proportion of collected TV sets began to decline gradually and dropped to 70.25% by 2015. Conversely, the proportion of the other four categories began to rise after 2014. The personal computer had the fastest increase, followed by the washing machine. Such changes indicate that the WEEE treatment fund has made great progress in diverting the flow direction of different kinds of WEEE.

• Collection rate

The total quantity of WEEE and the collection rate were calculated according to the procedures described above, and these data are shown in Table 4 and Table 5.

Table 5 shows that the collection rates from 2012 to 2015 increased year by year, and the collected amounts of equipment increased, as well. These results prove that the goal of the WEEE treatment fund was achieved to a certain extent in regulating the flow direction of WEEE in waste pool. Second, the collection rates of the refrigerator, washing machine, air-conditioner, and personal computer were still fairly low compared with the rate of the TV set. These results occurred because these items were only collected in small amounts.

#### 3.1.2. Treatment Target

• Normative recycled quantity

**Figure 4:**
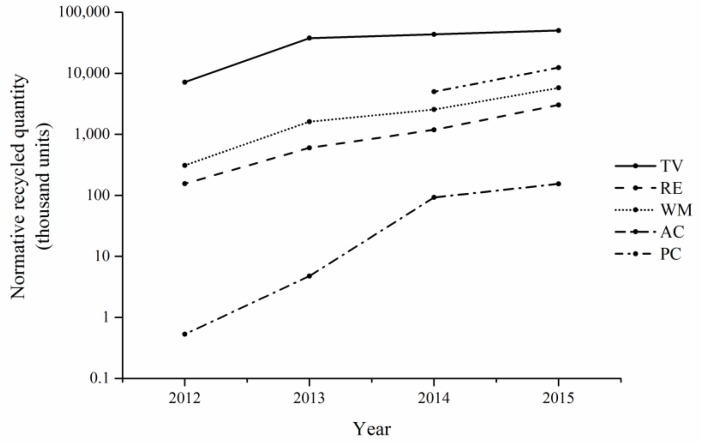
The normative recycled quantities of five categories of WEEE from 2012 to 2015.

The normative recycled quantities of five categories of WEEE are illustrated in Figure 4.

**Figure 5:**
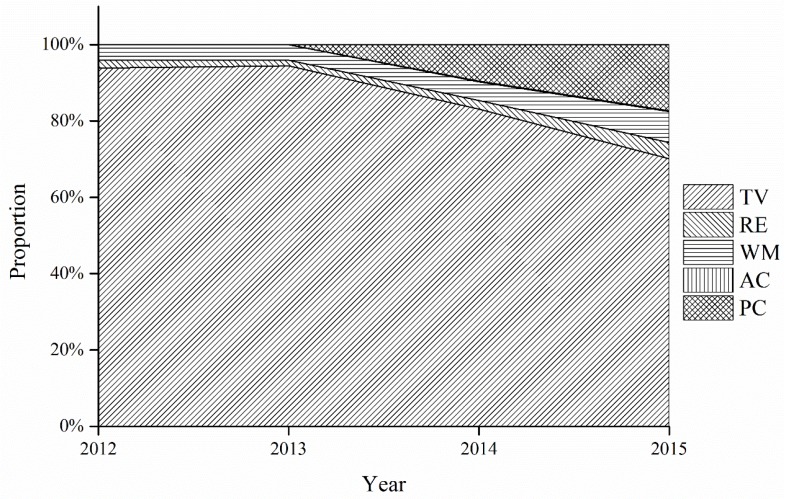
The proportion of five categories of WEEE in the total recycled quantities from 2012 to 2015.

Figure 4 shows that the treatment scale of five categories in the authorized enterprises continuously expanded from 2012 to 2015. The comparison of the indicator values among the five categories of WEEE is shown in Figure 5. Figure 5 indicates that the proportion of the normative recycled quantities of five categories of WEEE in total quantities all remained relatively stable before 2014. The TV set ranked first, and the air-conditioner came last. There was also an evident disparity between the TV set and the other four categories of WEEE. Despite the TV set’s dominance, the overall recycling of TV set began to experience a downward trend beginning in 2014. Compared to the proportion of TV set in 2012, the proportion in 2014 and 2015 decreased by 10.75% and 23.74%, respectively, while the proportion of the other four categories increased from 2012 to 2015. The personal computer experienced the largest increase, 17.34%. It seems that the recycling of the TV set no longer holds absolute dominance over the other four categories.

• Normative recycling rate

**Figure 6:**
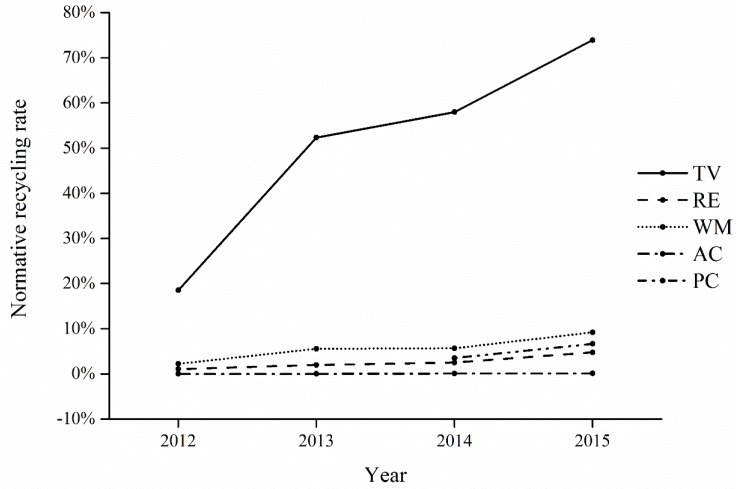
The normative recycling rates of five categories of WEEE from 2012 to 2015.

The results shown in Figure 6 indicate that the normative recycling rates of five categories of WEEE all increased significantly from 2012 to 2015, after the implementation of the WEEE treatment fund. The ratios of five categories of WEEE recycled normatively increased. In addition, the gap between the collected quantity and normative recycled quantity narrowed gradually, which led to the situation where the values of the collection rate were very close to those of the normative recycling rate in 2015.

• Recycling capacity rate

The results of the recycling capacity rate are summarized in Table 6. The results indicate that the recycling capacity rates increased from 10.17% in 2012 to 46.67% in 2015 in parallel with the increasing amount of WEEE recycled by the authorized enterprises. Although the values of this indicator were still less than 50%, the capacity rates have maintained a general uptrend. This result reflects the fact that the authorized enterprises were becoming more competitive in collecting and recycling WEEE, which means that the enterprises have found a way out of the predicament caused by the lack of materials. The WEEE treatment fund helped the authorized enterprises achieve sustainable development to some extent.

### 3.2. Performance Aspect

#### 3.2.1. Resource Efficiency

• Recovered resources

**Figure 7:**
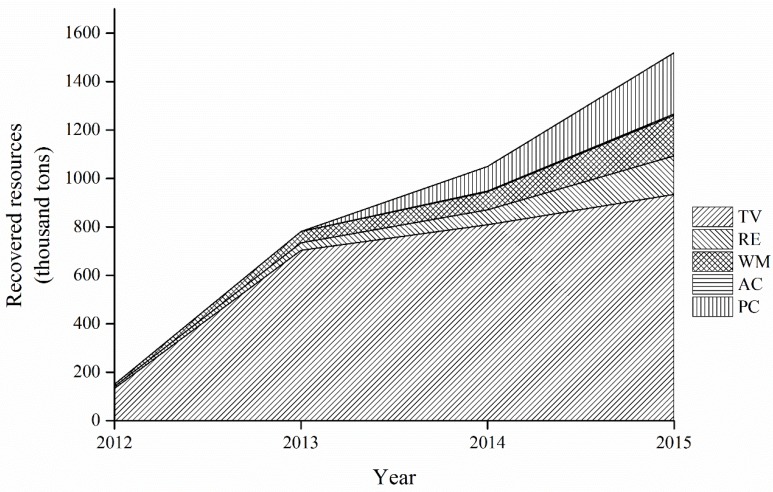
The recovered resources of five categories of WEEE from 2012 to 2015.

The recovered resources gained from the recycling processes of five categories of WEEE were calculated and presented in Figure 7.

Figure 7 illustrates the following points. First, the recovered resources of five categories show an increase from 150.13 thousand tons in 2012 to 1519.27 thousand tons in 2015, a 9-fold increase. It is particularly noticeable that the WEEE treatment fund played an increasingly significant role in improving resource efficiency. Second, the resources recovered from the recycling processes were mainly contributed by the TV set, the proportion of which was more than 60%. The proportions of the remaining four categories of WEEE were, in descending order: personal computer, washing machine, refrigerator, and air-conditioner. Third, the proportion of each category of WEEE in the total quantity changed little before 2014. However, the proportion of TV sets, which was originally the largest, began to decrease in 2014. The cause of this decline may lie in the fact that the recovered quantity of TV sets was the smallest, so the share of the TV set would be on the decline immediately if the WEEE treatment fund has the same promoting effect on the five categories of WEEE.

• Recovered resource rate

Table 7 shows the results of the recovered resource rates for five categories of WEEE. The following conclusions can be drawn from Table 7. The recovered resource rates of five categories all assumed an ascending tendency, which confirmed the successful performance of the WEEE treatment fund in resource conservation. The TV set had an edge in regards to the collected quantity and normative recycled quantity, so it far outstripped the other four categories of WEEE in reaping the resource benefits.

#### 3.2.2. Environmental Performance

The LCIA results are listed in Table 8.

Table 8 shows that the values for the environmental performance of collecting and recycling one unit of TV set, refrigerator, washing machine, air-conditioner, and personal computer in the informal sectors are 1.10 times, 22.73 times, 1.01 times, 145.08 times, and 1.10 times as much as those of the authorized recyclers, respectively. These data demonstrate that the authorized recyclers exhibited a much better environmental performance than the informal recyclers. In particular, when we compared the results of the refrigerator and air-conditioner to the other three categories of WEEE, the difference between the authorized and informal recyclers became more pronounced. The fundamental cause of this result may lie in the refrigerant typically found in the refrigerator and air-conditioner, which can cause severe environmental problems. In recycling activities, the authorized recyclers always treated the refrigerant as a kind of hazardous waste and disposed of it in a professional way, while the majority of the informal recyclers usually did nothing other than dump it in the environment. On the contrary, there are few pollutants that need special treatment like the refrigerant in the other three categories and the processes themselves require little professional operation. Therefore, the differences in the environmental performance between the authorized and informal recyclers are subtle.

In summary, the WEEE treatment fund has already achieved significant environmental benefits. The environmental impact can be reduced as long as the authorized enterprises collect and recycle one more unit of TV set, refrigerator, washing machine, air-conditioner, and personal computer, and these reductions would be 10.28%, 2172.77%, 1.18%, 14,408.42%, and 9.84%, respectively.

## 4. Conclusions

We assessed the effectiveness of the WEEE treatment fund. According to the assessment results, we concluded that the WEEE treatment fund has both achievements and problems, and this was especially true during the initial stage of its implementation.

In conclusion, first of all, the WEEE treatment fund made progress in achieving the goal of promoting collection and recycling activities in regulated channels from 2012 to 2015. Secondly, the WEEE treatment fund has produced significant benefits with regards to resources and the environment. However, the differences in effectiveness among the five categories of WEEE were observed and the collection and recycling of TV sets performed best. Although the promotional effects of the WEEE treatment fund on four categories of WEEE (excluding the TV set) were increasingly significant, the unbalanced situation, caused by the characteristics of the recycled products, market structure, and insufficient subsidies, remains. Among them, the subsidy rate is the key factor in the effectiveness of the WEEE treatment fund.

At present, the administration has already adjusted the unreasonable subsidy rates and put them in force in 2016. Considering the decisive role subsidies play in the success of the WEEE treatment fund, this measure was necessary and timely. However, the rationality and the effectiveness of the new subsidy rates should also be assessed in detail as soon as possible. Besides, from the perspective of management, there have been problems with the WEEE treatment fund’s fiscal balance [14], supervision, and general approach. To further optimize the effectiveness of the WEEE treatment fund, additional research should be carried out to determine the scope of, and solutions to, such issues. Furthermore, this research mainly focused on the achievement of the general goal of the WEEE treatment fund, but in consideration that the producers who pay for the subsidies will undoubtedly be affected, the relevant assessment should also be carried out.

## Figures and Tables

**Table 1:** The surcharge rates and subsidy rates of EEE (Unit: yuan RMB/unit).

Category		Surcharge Rate	Subsidy Rate	
			Old	New
TV	14 inches ≤ Size < 25 inches	13	85	60
	Size ≥ 25 inches			70
RE	50 L ≤ Volume ≤ 500 L	12	80	80
WM	Single-tub, Dryer (3 kg < Capacity ≤ 10 kg)	7	35	35
	Double-tub, Vertical axis, Drum-type (3 kg < Capacity ≤ 10 kg)			45
AC	Refrigerating capacity ≤ 14,000 W	7	35	130
PC	-	10	85	70

Sources: Regulations on the Collection and Use of the WEEE Treatment Fund (2012) and the WEEE surcharge rates and subsidy rates (2015).

**Table 2:** The indicators of the effectiveness assessment of the WEEE treatment fund.

Category		Indicators	Matrix *
Processing aspect	Collection target	Collected quantity	$C_{n}$
		Collection rate	$R_{c,n}=C_{n}/Q_{n}^{\prime }$
	Treatment target	Normative recycled quantity	$D_{n}$
		Normative recycling rate	$R_{r,n}=D_{n}/Q_{n}^{\prime }$
		Recycling capacity rate	$U_{n}=D_{n}/P_{n}$
Performance aspect	Resource efficiency	Recovered resources	$R_{n}=\sum R_{\mathrm{in}}=\sum \left(f_{i}\times \bar{w}\times D_{n}\right)$
		Recovered resource rate	$l_{n}=\frac{R_{n}}{Q_{n}^{\prime \prime }}=\frac{R_{n}}{\bar{w}\times Q_{n}^{\prime }}$
	Environmental performance	Environmental performance	$\Delta E=\left(E_{w}-E_{f}\right)/E_{f}$

* Where Q_n_′ is the total quantity of WEEE; P_n_ is the recycler capacity; R_in_ is the recovered quantity of resource *i*; f_i_ is the recovered rate for resource *i* from the given WEEE; $\bar{w}$ is the average weight of the given WEEE; Q_n_″ is the total weight of the WEEE; E_w_ is the environmental performance of informal recyclers; E_f_ is the environmental performance of authorized recyclers; and n is a given year.

**Table 3:** Life cycle inventory (LCI) data sources.

Type	Category	Source	Characteristic
Recycling process data	Average distance of transportation	A WEEE recycling enterprise in Hubei Province	Site-specific data
	Composition of EEE	An environmental impact report from a WEEE recycling enterprise in Shanghai; from the literature [33,34,35,36]	Secondary data
	Disassembly	Environmental impact reports/ technical evaluation reports from several WEEE recycling enterprises; CAS RCEES * 2012; Ecoinvent database	
	Recycling		
	Final disposal		
Basic data	Transportation system	CAS RCEES 2012	
	Energy system		
	Basic material production	CAS RCEES 2012; Ecoinvent database	
	Waste management	Ecoinvent database	

* CAS RCEES refers to the underlying database developed by Research Center for Eco-Environmental Sciences, Chinese Academy of Sciences.

**Table 4:** The total quantities of five categories of WEEE from 2012 to 2015 (in thousands of units).

Category	2012	2013	2014	2015
TV	38,503	71,934	74,746	67,745
RE	14,603	30,444	47,086	63,634
WM	13,922	29,031	44,974	62,484
AC	28,244	62,451	101,716	144,455
PC	43,198	91,825	141,993	186,295

**Table 5:** The collection rates of five categories of WEEE from 2012 to 2015.

Category	2012	2013	2014	2015
TV	22.15%	53.37%	58.14%	74.97%
RE	1.10%	1.98%	2.56%	4.83%
WM	2.39%	5.57%	5.68%	9.32%
AC	0.003%	0.01%	0.09%	0.11%
PC	-	-	3.51%	6.69%

**Table 6:** The recycling capacity rates from 2012–2015.

Category	2012	2013	2014	2015
Number of enterprises (unit)	42	91	106	109
Processing capability (thousand units)	74,679.36	136,558.44	151,537.54	152,997.54
Enterprise capacity utilization rate	10.17%	29.20%	34.41%	46.67%

**Table 7:** The recovered resource rates of five categories of WEEE from 2012 to 2015.

Category	2012	2013	2014	2015
TV	13.65%	38.59%	42.75%	54.50%
RE	0.93%	1.72%	2.19%	4.15%
WM	2.12%	5.30%	5.41%	8.78%
AC	0.002%	0.01%	0.09%	0.10%
PC	-	-	2.82%	5.34%

**Table 8:** Life cycle impact assessment (LCIA) endpoint assessment results (Unit: Pt).

Category	TV	RE	WM	AC	PC
Authorized	0.246	0.813	0.309	0.563	0.278
Informal	0.272	18.485	0.312	81.656	0.305

## Supplementary Materials

The following are available online at http://www.mdpi.com/1660-4601/15/5/1028/s1, Table S1: The recovery rates of five categories of WEEE, Table S2: The average weight of five categories of WEEE (Unit: kg), Table S3: The lifespan distribution of five categories of WEEE, Table S4: The sales of five categories of EEE (in thousands of units), Table S5: The theoretical estimation of five categories of WEEE from 2012 to 2015 (in thousands of units), Table S6: The recycling process data for one unit of TV set and personal computer, Table S7: The recycling process data for one unit of refrigerator, Table S8: The recycling process data for one unit of washing machine, Table S9: The recycling process data for one unit of air-conditioner.
