# Supplementary material for: An Effectiveness Assessment of China’s WEEE Treatment Fund
Source: Int J Environ Res Public Health. 2018 May 19;15(5):1028. doi: 10.3390/ijerph15051028 (PMC5982067; doi:10.3390/ijerph15051028)
Supplement: Supplementary file 1 [file ijerph-15-01028-s001.pdf]

An Effectiveness Assessment of China's WEEE Treatment Fund

Wenyan Zhao, Jianxin Yang

Supplementary Materials

Table S1. The recovery rates of five categories of WEEE.

Disassembled product	TV	RE	WM	AC	PC
Iron and its alloys	7.05%	45.69%	54.29%	57.86%	34.12%
Copper and its alloys	4.30%	3.66%	4.08%	20.48%	3.20%
Aluminum and its alloys	-	0.97%	1.02%	3.99%	-
Nickel	0.01%	0.004%	0.002%	0.003%	0.02%
Gold	-	-	-	-	0.0003%
Lead-free glass	43.62%	17.90%	5.08%	-	24.68%
Plastic	18.75%	18.97%	30.89%	14.12%	18.36%
Solid waste	26.28%	12.81%	4.64%	3.54%	19.62%
Total	100.00%	100.00%	100.00%	100.00%	100.00%

Table S2. The average weight of five categories of WEEE (Unit: kg).

Category	TV	RE	WM	AC	PC
Average weight	25.31	60.00	30.50	43.20	25.36

Table S3. The lifespan distribution of five categories of WEEE.

Category	Lifespan (year)	Lifespan distribution (%)					
TV	8-10	11.12(8)	38.88(9)	38.88(10)	11.12(11)	-	-
RE	8-12	7.93(8)	15.96(9)	26.11(10)	26.11(11)	15.96(12)	7.93(13)
WM	8-12	7.93(8)	15.96(9)	26.11(10)	26.11(11)	15.96(12)	7.93(13)
AC	7-11	7.93(7)	15.96(8)	26.11(9)	26.11(10)	15.96(11)	7.93(12)
PC	2-6	7.93(2)	15.96(3)	26.11(4)	26.11(5)	15.96(6)	7.93(7)

* The numbers in the brackets represent the lifespan.

Table S4. The sales of five categories of EEE (in thousands of units).

Year	TV	RE	WM	AC	PC
1999	-	13,764.97	12,805.45	-	-
2000	-	12,871.68	13,457.76	14,682.23	-
2001	31,269.87	12,261.75	11,846.77	17,600.59	-
2002	35,838.34	13,657.40	13,743.80	23,328.99	-
2003	41,661.22	18,410.70	16,069.86	31,794.90	-
2004	44,007.08	22,083.76	19,066.20	40,600.77	-
2005	41,954.25	14,201.13	20,877.63	42,843.53	34,036.42
2006	28,325.60	15,811.88	24,216.02	42,223.41	33,968.63
2007	38,127.44	21,879.91	26,683.95	48,173.94	39,559.57
2008	42,963.85	-	-	49,572.41	50,433.45
2009	-	-	-	-	51,207.06
2010	-	-	-	-	42,975.76

2011	-	-	-	-	78,455.87
2012	-	-	-	-	23,576.25
2013	-	-	-	-	20,802.10

Table S5. The theoretical estimation of five categories of WEEE from 2012 to 2015 (in thousands of units).

Category	2012	2013	2014	2015
TV	38,502.63	41,958.37	41,204.30	36,458.17
RE	14,602.96	16,001.40	17,245.56	17,754.02
WM	13,921.73	15,440.85	17,559.38	20,064.88
AC	28,243.58	34,207.68	39,270.81	42,831.85
PC	43,198.28	48,626.24	50,168.54	49,290.19

Table S6. The recycling process data for one unit of TV set and personal computer.

	Category	TV	PC
Input	Electricity (kW·h)	0.29	0.17
Output	General metal (kg)	2.68	8.96
	Glass (kg)	16.44	9.35
	Plastic (kg)	4.55	4.34
	Printed Circuit Board (kg)	1.26	1.99
	Cable (kg)	0.28	0.45
	Solid waste (kg)	0.08	0.05
	Fluorescent powder (kg)	0.02	0.02
	Dust (kg)	0.001	0.001

Table S7. The recycling process data for one unit of refrigerator.

	Category	Value
Input	Electricity (kW·h)	1.64
Output	Glass (kg)	10.74
	General metal (kg)	29.94
	Plastic (kg)	11.04
	Printed Circuit Board (kg)	0.45
	Cable (kg)	0.48
	Dust (kg)	0.002
	Refrigerant (kg)	0.15
	Lubricant (kg)	0.34
	Rubber (kg)	0.85
	PUR foam (kg)	6.00

Table S8. The recycling process data for one unit of washing machine.

	Category	Value
Input	Electricity (kW·h)	0.03
Output	General metal (kg)	17.96
	Glass (kg)	1.55
	Cable (kg)	0.40
	Printed Circuit Board (kg)	0.15
	Plastic (kg)	9.14
	Solid waste (kg)	1.30

Table S9. The recycling process data for one unit of air-conditioner.

Category		Value
Input	Electricity (kW·h)	0.12
	General metal (kg)	28.96
	Cable (kg)	1.80
	Printed Circuit Board (kg)	0.30
Output	Plastic (kg)	4.84
	Condenser (kg)	6.00
	Refrigerant (kg)	0.80
	Lubricant (kg)	0.50
